# Discharge Hemoglobin Association with Long-Term Outcomes of ST-Elevation Myocardial Infarction Patients Undergoing Primary Percutaneous Coronary Intervention

**DOI:** 10.1155/2020/8647837

**Published:** 2020-02-27

**Authors:** Ming Gao, Xinying Zhang, Ling Qin, Yang Zheng, Zhiguo Zhang, Qian Tong, Hang Li

**Affiliations:** Cardiovascular Center, The First Hospital of Jilin University, Changchun 130021, China

## Abstract

**Background:**

Anemia following acute myocardial infarction (AMI) is associated with poor outcomes. While previous studies in patients with AMI have focused on anemia at admission, we hypothesized that hemoglobin (Hb) decline during hospitalization and lower discharge Hb would be associated with greater long-term mortality in patients undergoing primary percutaneous coronary intervention (PCI) for ST-segment elevation myocardial infarction (STEMI).

**Methods:**

We analyzed records of 983 STEMI patients who were treated with primary PCI. The primary end point was all-cause mortality at 1 year and 2 years. The relationship between discharge Hb levels, decline in Hb levels, bleeding event classification, and all-cause mortality was determined.

**Results:**

Overall, 16.4% of patients had bleeding events, which were classified by the Thrombolysis in Myocardial Infarction (TIMI) score as 7% minimal, 8.6% minor, and 0.9% major. No significant gastrointestinal bleed and cerebral hemorrhage occurred in hospitals among these patients. The incidence rate of the 2-year all-cause mortality increased with severity of the bleeding event score (8.78% for no bleeding vs. 11.59% for minimal bleeding vs. 20.24% for minor bleeding vs. 55.56% for major bleeding, *P* < 0.001). Discharge Hb was significantly associated with 2-year mortality in an unadjusted model (hazard ratio (HR) per 1 g/L decrease in discharge Hb = 1.020, 95% confidence interval (CI): 1.006–1.034, *P* < 0.001). Discharge Hb was significantly associated with 2-year mortality in an unadjusted model (hazard ratio (HR) per 1 g/L decrease in discharge Hb = 1.020, 95% confidence interval (CI): 1.006–1.034, *P* < 0.001). Discharge Hb was significantly associated with 2-year mortality in an unadjusted model (hazard ratio (HR) per 1 g/L decrease in discharge Hb = 1.020, 95% confidence interval (CI): 1.006–1.034,

**Conclusions:**

In this population of patients hospitalized for STEMI, all-cause mortality increased with lower discharge Hb, and discharge Hb was a significant predictor of mortality risk.

## 1. Introduction

Anemia following acute myocardial infarction (AMI) is associated with poorer outcomes relative to nonanemic AMI patients. Furthermore, anemia is more frequently encountered in patients hospitalized for cardiac events than in the general population [1, 2], ranging from 11% to 38% in patients with AMI [3, 4].

During myocardial infarction, oxygen delivery to the heart is reduced. In AMI, the amount of oxygen delivered to the heart is further decreased, and this insufficient oxygenation jeopardizes the myocardium. After recovery, cardiac output is increased to maintain adequate systemic oxygen delivery, a compensation that might impair recovery. Indeed, previous studies show that anemic patients experience worse outcomes after percutaneous coronary intervention (PCI; e.g., increased risks for stent thrombosis, long-term mortality, and bleeding) than myocardial infarction patients without anemia [5, 6].

The majority of previous studies in patients with AMI have focused on anemia present at admission [6, 7], and little is known regarding hemoglobin (Hb) levels at discharge among patients who undergo primary PCI for ST-segment elevation myocardial infarction (STEMI) or the change in Hb levels and mortality among patients' subsequent outcomes. Because of the additional cardiac strain caused by anemia after STEMI, we hypothesized that Hb decline and lower discharge Hb would be associated with a steeper functional decline in patients undergoing primary PCI for STEMI.

## 2. Methods

### 2.1. Study Population

We conducted a retrospective analysis of a cohort of consecutive patients with STEMI who underwent primary PCI in the Cardiology Department at the First Hospital of Jilin University between January 1, 2014, and December 31, 2015. The demographic, clinical, and angiographic characteristics of the patients had been prospectively collected in the department's electronic medical records, which store all patient-related health information obtained at primary care. Diagnosis of STEMI was consistent with the European Society of Cardiology/American College of Cardiology consensus document [8]. Patients who met at least 2 of the following criteria were included: characteristic severe chest pain or other symptoms suggestive of ischemia, electrocardiographic changes, and/or cardiac troponin value above the ninety-ninth percentile reference limits. A total of 1,615 STEMI patients who underwent emergency primary PCI were examined and, after excluding those who received emergency coronary artery bypass graft surgery, were diagnosed with thrombolysis or given only medical treatment or had unavailable Hb data at baseline and before discharge; 983 consecutive patients were included in our study. No significant gastrointestinal bleed and cerebral hemorrhage occurred in hospitals among these patients, and gastrointestinal bleed and cerebral hemorrhage were implicitly excluded from this study because patients with bleeding events are less likely to undergo PCI. The primary end point of the study was the occurrence of death. Discharge Hb was a covariate variable. In-hospital bleeding episodes were classified using the Thrombolysis in Myocardial Infarction (TIMI) criteria [9]. TIMI major bleeding was defined as intracranial hemorrhage or a decrease in Hb from admission to discharge greater than or equal to 5 g/dL. Minor bleeding was defined as a 3–5 g/dL decline in Hb, and minimal bleeding was defined as a less than 3 g/dL decline in Hb.

The study protocol was approved by the Ethics Review Board of the First Hospital of Jilin University (No. 2016-263), and all participating patients approved the research protocols.

### 2.2. Laboratory Data

Peripheral blood samples were obtained at the time of hospital admission prior to performing primary PCI. The blood samples were used for routine blood chemistry and blood counts such as hemoglobin, total cholesterol (TC), creatinine, and triglyceride (TG) at the certified laboratory department of the First Hospital of Jilin University. The primary predictor variable for all analyses was Hb data following admission and before discharge. Discharge Hb was defined as the last Hb value (g/dL) obtained within 48 hours of discharge from the hospital.

### 2.3. Protocol and Definition

All patients were pretreated with a loading dose of 300 mg aspirin, 600 mg clopidogrel, and 1000 IU/L intravenous unfractionated heparin. Coronary angiography was performed using standard techniques. Thrombus aspiration, stent type, predilation, postdilation, and placement of a temporary pacemaker or intra-aortic balloon pump were decided at the surgeon's discretion. Following PCI, dual antiplatelet therapy (DAPT) with aspirin (100 mg/day) and clopidogrel (75 mg/day) was recommended in all patients with successful implantation of drug-eluting stents. Current American College of Cardiology/American Heart Association (ACC/AHA) [10] and European Society of Cardiology (ESC) guidelines [11] recommend DAPT of aspirin plus a P2Y12 inhibitor for at least 12 months or longer after implantation of drug-eluting stents in patients with acute coronary syndrome. During the time frame in which our study was conducted, our center recommended 18 months of DAPT.

### 2.4. Follow-Up

The patients' clinical courses were monitored by telephone interviews every six months after discharge and a comprehensive review of electronic medical records from our outpatient clinic. All patients were monitored over a period of 2 years or until death whichever occurred first. In the case of patients who were lost to follow-up, we contacted the patients' families or used the Social Security Administration Death Master File to confirm mortality.

### 2.5. Statistical Analysis

Characteristics of groups with or without Hb decline at baseline were compared by chi-squared tests, independent-group *t*-tests, and Wilcoxon rank-sum tests, depending on the variable levels and distribution of measurements after testing for normality by the Kolmogorov–Smirnov test. The missing rates were less than 5% for each variable, and the attribute values were missing at random. The default missing-data analysis was complete-case analysis. Cox multiple regression analysis was performed to evaluate the independent contributions of baseline clinical characteristics, medical history, and laboratory data to the occurrence of all-cause mortality at 2 years with forward stepwise selection. Patients were grouped according to the quintile of discharge Hb to permit comparison of the relationship with 2-year outcomes between the groups (quintile 1: 5.4–11.3 g/dL; quintile 2: 11.3–12.5 g/dL; quintile 3: 12.5–13.5 g/dL; quintile 4: 13.5–14.6 g/dL; quintile 5: 14.6–18.8 g/dL). Hb levels of quintile 3 were used as a reference for odds ratio (OR) analyses. Logistic regression was performed to find whether a dose-response effect exists. Receiver-operating characteristic area under the curve (ROC-AUC) was constructed to further illuminate the best cutoff values per 1 g/L decrease in discharge Hb to predict 2-year all-cause mortality. Kaplan–Meier survival curve analysis compared survival among the severity of bleeding groups using the log-rank test. All analyses were conducted using Stata software, version 12 (Stata Corp., College Station, TX), and a two-sided *P* < 0.05 was considered statistically significant.

## 3. Results

### 3.1. Baseline Characteristics

**Figure 1:**
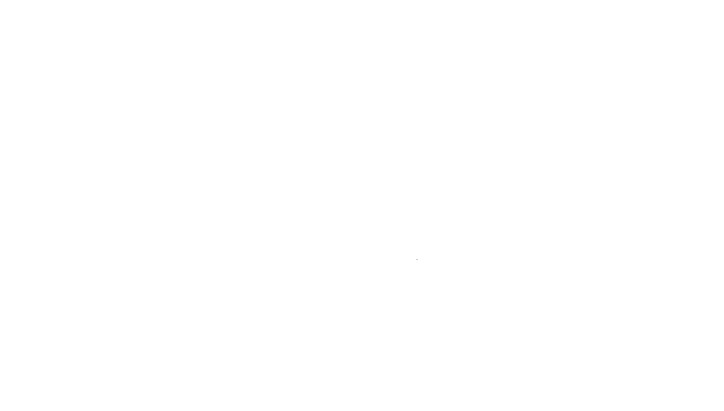
Flow chart of selection.

The baseline characteristics of the 983 patients in the study population included a mean age of 61 ± 12 years; 66.9% of the participants were men (Figure 1) (Table 1). The majority of patients (93.2%) underwent balloon angioplasty, and 86.9% received drug-eluting stent implantation. The mean admission hemoglobin was 14.1 ± 2.1 g/dL, and the mean discharge hemoglobin was 12.9 ± 2.0 g/dL. Overall, 16.4% of patients had bleeding events, classified under TIMI scores as 7.0% minimal, 8.6% minor, and 0.9% major. Among the total of nine patients (0.9%) who had TIMI major bleeding events, four patients died before discharge and the remaining five experienced gradual reductions in Hb (the lowest of which was >9.3 g/dL) without an additional significant clinical bleed in hospitals. Nonetheless, this group experienced an overall decline of health due to multiple concurrent diseases such as congestive heart failure, chronic renal failure, or pneumonia. Of the patients who died, prior to discharge, one case was complicated by acute renal insufficiency and one by the combination of pneumonia, acute respiratory failure, and ventricular arrhythmia. For both of these patients, Hb fluctuated between 7.4 and 8.7 g/dL after surgery but was lowest (5.4 g/dL) on the day of death. The other two patients who died after surgery had Hb values greater than 9.0 throughout the postsurgical period. None of these four patients received transfusion therapy.

Because pharmacological therapy could indeed have affected discharge Hb values, we controlled for pharmacological factors in the statistical analysis (Table 1). Aspirin, clopidogrel, and heparin therapy was initiated in all STEMI patients. The glycoprotein IIb/IIIa inhibitor tirofiban hydrochloride was administrated in patients with serious, angiography-proven thrombus burden. There were no differences between two groups in in-hospital pharmacological therapy.

### 3.2. Association between Bleeding and Mortality

**Figure 2:**
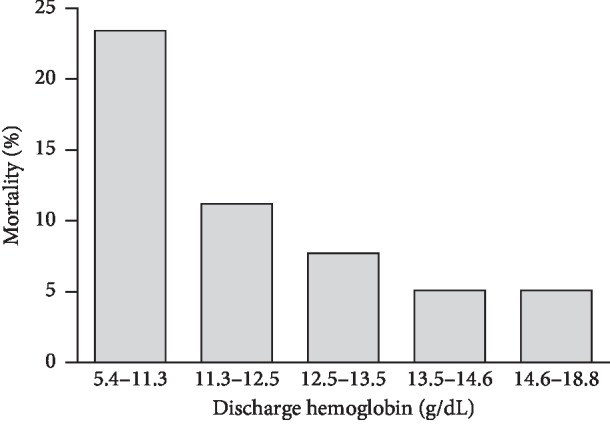
The incidence rate of unadjusted 2-year all-cause mortality was significantly higher for patients with lower discharge Hb.

**Figure 3:**
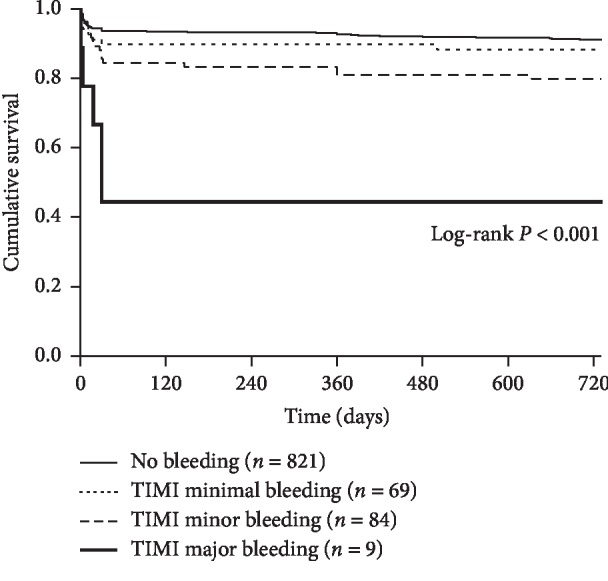
Kaplan–Meier survival curve analysis comparing cumulative incidence of all-cause mortality according to severity of bleeding events by Thrombolysis in Myocardial Infarction (TIMI) classification.

During the 2-year follow-up period, all-cause mortality was 8.96% (89 deaths) at 1 year and 10.39% (103 deaths) at 2 years. There was a negative relation between survival and anemia severity. The 2-year all-cause mortality was significantly higher along with lower discharge Hb quintiles (*P* < 0.001, Figure 2). In Kaplan–Meier survival curve analysis, the incidence rate of 2-year all-cause mortality was significantly higher for TIMI minor and major bleeding events than for patients with no bleeding events (8.78% for no bleeding vs. 11.59% for TIMI minimal bleeding vs. 20.24% for TIMI minor bleeding vs. 55.56% for TIMI major bleeding, *P* < 0.001, Figure 3).

### 3.3. Association between Discharge Hb and Mortality

**Figure 4:**
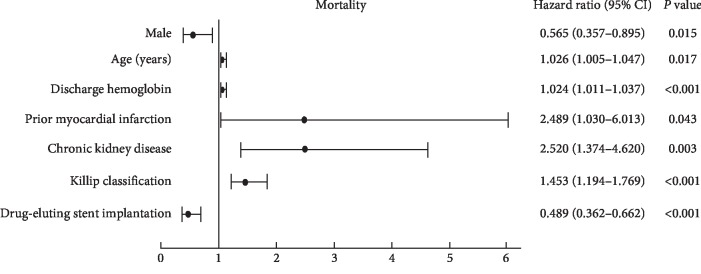
Multivariate predictors of 2-year mortality. CI, confidence interval.

According to Cox proportional-hazards regression models, discharge Hb was significantly associated with 2-year mortality in the unadjusted model (hazard ratio (HR) per 1 g/dL decrease in discharge Hb = 1.020, 95% CI: 1.006–1.034, *P*=0.004) and remained significant after adjustment for potential confounders (HR per 1 g/dL decrease in discharge Hb = 1.024, 95% CI: 1.011–1.037, *P* < 0.001). In multivariable models adjusting for patients' demographic and clinical characteristics, discharge Hb, older age, female sex, prior myocardial infarction, chronic kidney disease, Killip classification >2, and absence of drug-eluting stent implantation were independent predictors for 2-year mortality (Figure 4).

### 3.4. Hb Stratification and Clinical Outcomes

The OR for all-cause mortality at 2 years for participants with discharge Hb below the twentieth percentile was 3.529 (95% CI: 1.976–6.302) and 2.968 (95% CI: 1.614–5.456) after adjustment for age and sex and 2.485 (95% CI: 1.310–4.715) after adjustment for all covariables (Table 2). Using a 2.6 g/dL decline in Hb as a cutoff for ROC-AUC, discharge Hb predicted 2-year mortality with a sensitivity of 86.55% and specificity of 80.16% (ROC-AUC: 0.550, 95% CI: 0.484–0.625, *P*=0.034).

## 4. Discussion

In this population of patients hospitalized for STEMI, all-cause mortality increased with severity of bleeding events, and discharge Hb was a statistically significant predictor of mortality risk.

Published studies focus on the relationship between anemia and outcomes in patients presenting with acute coronary syndromes and/or undergoing revascularization, demonstrating that anemia measured at a single time point is associated with worse outcomes [12–14]. In contrast, few studies have evaluated patients with STEMI. Therefore, the aim of the current analysis was to assess the relationship between discharge Hb and decline in Hb between admission and discharge with long-term, all-cause mortality in patients undergoing PCI for STEMI.

Anemia significantly decreases oxygen delivery to the myocardium downstream of coronary stenosis and increases myocardial oxygen demand by necessitating a higher stroke volume and heart rate to maintain adequate systemic oxygen delivery [15–17]. In STEMI, even mildly reduced Hb concentrations at the abrupt onset of coronary occlusion may significantly attenuate the ability of collateral flow from nearby patent vessels to limit the extent of myocardial necrosis and peri-infarct ischemia. The combination of these processes may explain the pathophysiology underlying the progressively poorer outcomes we observed in more severe bleeding events and lower discharge Hb concentrations. Furthermore, discharge Hb is an elegant marker for prognosis because it is routinely measured in patients hospitalized with AMI, and it is potentially modifiable with treatment. Notably, many patients in our cohort (16.4%) had some decline in their Hb during AMI admission which is a high figure than expected.

Furthermore, we subdivided patients according to discharge Hb and found increased mortality risks in patients with only mildly reduced Hb concentration and a dose-response effect that resulted in progressively lower survival rates with more profound degrees of anemia. These effects may have been masked in previous studies, which generally only distinguish between patients with and without anemia. In a meta-analysis of 233,144 patients with acute coronary syndromes, anemia was associated with a significantly increased risk of death or reinfarction [14]. In a study of 936 women undergoing evaluation for chest pain, Hb was an independent predictor of adverse cardiovascular outcomes, with a 20% increased risk for each 1 g/dL decrement in Hb [18]. Furthermore, in the elderly, per 1.0 g/dL decrease in Hb over 3 years was associated with a 1.11-fold increased mortality over subsequent follow-up [19]. Although we demonstrated that the impact of Hb decline on mortality was greatest at lower discharge Hb concentrations, any decline greater than 2.6 g/dL between admission and discharge conferred an increased risk of 2-year mortality.

Thus, prior research and our study demonstrate that both the absolute level of Hb and the change in Hb during hospitalization are important when evaluating the association of Hb concentrations with mortality. Prior treatment strategies to increase Hb concentration in patients with AMI and anemia might not significantly benefit long-term survival because guidelines do not specify Hb targets. Current clinical practice guidelines regarding blood transfusion are almost always indicated if Hb < 6 g/dL and rarely indicated if Hb > 10 g/dL [20]. For patients with Hb 6–10 g/dL, transfusion decisions depend on the extent of the blood loss, underlying cardiovascular disease, and overall clinical status. The state of anemia may represent an overall decline of health due to underlying comorbidities, and a simple preoperative correction of hemoglobin via transfusion may not positively affect the patient's operative course. However, other strategies can reduce perioperative risk in anemic patients, including the use of certain antiplatelet agents with care to avoid bleeding, using proton pump inhibitors to minimize upper gastrointestinal bleeding, and avoiding the use of glycoprotein IIb/IIIa inhibitors. Given these findings, a prospective randomized clinical trial may be warranted to determine whether precise targeting of Hb levels improves outcomes in patients with AMI. Indeed, further study is necessary to assess whether intervening to increase Hb (outside of treating nutritional deficiencies), addressing blood loss, and managing chronic disease have any role in ameliorating adverse mortality outcomes after STEMI.

### 4.1. Study Limitations

There are several limitations to our study. Discharge hemoglobin was not routinely captured in our center. We excluded 36% of patients who did not have complete evaluation of discharge hemoglobin. The results may be biased due to the potential impact of data that were not available in our database, although there was continuous admission. Even though important comorbidities were included in our analysis, data on other measures of the overall health status (e.g., body mass index) were not available; thus, anemia may represent an overall decline of health due to underlying comorbidities. Despite the absence of this information, all categories of bleeding events and low discharge Hb predicted an increased risk for long-term mortality independent of a number of confounders in our study population. Prasugrel or ticagrelor was not available during the study period in our center, which prevents us from analyzing the impact of different P2Y12 inhibitors on Hb levels. We did not test routinely for drug use during follow-up; thus, the extent of adherence to the prescribed medical therapies could affect clinical outcomes, potentially causing us to overestimate mortality risk in patients with anemia. However, although anemia affected mortality, we cannot solely attribute the significantly worse long-term outcomes in patients with AMI to it.

## 5. Conclusions

In this population of patients hospitalized for STEMI, all-cause mortality increased when discharge Hb was low and when bleeding events during hospitalization were more severe; lower discharge Hb was associated with long-term, all-cause mortality in patients undergoing PCI for STEMI.

## Figures and Tables

**Table 1:** Baseline characteristics of participants (overall and by presence/absence of bleeding events).

Characteristics	Overall (*n* = 983), *n* (%) or mean ± SD	Without Hb decline (*n* = 821), *n* (%) or mean ± SD	Hb decline (*n* = 162), *n* (%) or mean ± SD	*P* value (without Hb decline vs. Hb decline)
Age (years)	61 ± 12	61 ± 12	62 ± 11	0.470
Male gender	658 (66.9)	551 (67.1)	107 (66.0)	0.793
Medical history				
Diabetes mellitus	251 (25.5)	209 (25.5)	42 (25.9)	0.900
Hypertension	462 (47.0)	384 (46.8)	78 (48.2)	0.748
Previous PCI	33 (3.4)	27 (3.3)	6 (3.7)	0.789
Atrial fibrillation	65 (6.6)	50 (6.1)	15 (9.3)	0.138
Peripheral vascular disease	9 (0.9)	4 (0.5)	5 (3.1)	0.008
Prior myocardial infarction	34 (3.5)	28 (3.4)	6 (3.7)	0.852
Prior cerebrovascular event	55 (5.6)	40 (4.9)	15 (9.3)	0.026
Chronic kidney disease	45 (4.6)	36 (4.4)	9 (5.6)	0.515
Arrhythmia (VT/VF)	82 (8.3)	65 (7.9)	17 (10.5)	0.278
Infarct location by electrocardiogram				0.269
Inferior	285 (29.0)	230 (28.0)	55 (34.0)	
Anterior	466 (47.4)	392 (47.8)	74 (45.7)	
Lateral	232 (23.6)	199 (24.2)	33 (20.4)	
Killip classification				0.018
I	613 (62.4)	522 (63.6)	91 (56.2)	
II	205 (20.9)	175 (21.3)	30 (18.5)	
III	52 (5.3)	39 (4.8)	13 (8.0)	
IV	113 (11.5)	85 (10.4)	28 (17.3)	
Laboratory data				
Total cholesterol (mmol/L)	4.7 ± 1.2	4.7 ± 1.2	4.8 ± 1.4	0.123
Triglycerides (mmol/L)	1.7 ± 1.1	1.7 ± 1.1	1.9 ± 1.3	0.211
Admission hemoglobin (g/dl)	14.1 ± 2.1	13.9 ± 2.1	15.0 ± 2.1	<0.001
Discharge hemoglobin (g/dl)	12.9 ± 2.0	13.1 ± 1.9	11.7 ± 2.1	<0.001
Method of reperfusion				0.897
Balloon angioplasty	916 (93.2)	767 (93.4)	149 (92.0)	
Drug-eluting stent implantation	854 (86.9)	714 (87.0)	140 (86.4)	
Thrombus aspiration	120 (12.2)	91 (11.1)	29 (17.9)	0.015
Temporary pacemaker	67 (6.8)	52 (6.3)	15 (9.3)	0.177
Number of stents	1.1 ± 0.7	1.1 ± 0.7	1.1 ± 0.7	0.626
Intra-aortic balloon pump	8 (0.8)	7 (0.9)	1 (0.6)	0.610
Aspirin	973 (99.0)	813 (99.0)	160 (98.8)	0.673
Clopidogrel	977 (99.4)	815 (99.3)	162 (100)	0.597
Tirofiban hydrochloride	112 (11.4)	95 (11.6)	17 (10.5)	0.787

SD, standard deviation; PCI, percutaneous coronary intervention; VT/VF, ventricular tachycardia/ventricular fibrillation.

**Table 2:** Association of discharge hemoglobin with 2-year all-cause mortality.

Hb concentration (g/dL)	Crude univariate model			Adjusted for age			Adjusted for all covariables		
	HR	95% CI	*P* value	OR	95% CI	*P* value	OR	95% CI	*P* value
Quintile 1: 5.4–11.3	3.529	1.976–6.302	<0.001	2.968	1.614–5.456	<0.001	2.485	1.310–4.715	0.005
Quintile 2: 11.3–12.5	1.418	0.731–2.750	0.302	1.347	0.687–2.641	0.385	1.220	0.604–2.465	0.580
Quintile 3: 12.5–13.5^a^	1 (ref)		<0.001	1 (ref)		<0.001	1 (ref)		<0.001
Quintile 4: 13.5–14.6	0.716	0.329–1.560	0.401	0.808	0.370–1.765	0.593	0.976	0.442–2.154	0.952
Quintile 5: 14.6–18.8	0.659	0.296–1.466	0.306	0.964	0.425–2.184	0.930	1.200	0.522–2.757	0.667

Hb, hemoglobin; HR, hazard ratio; OR, odds ratio; CI, confidence interval. ^a^Percentiles are shown in categories: quintile 1: <20^th^ percentile; quintile 2: 20^th^–<40^th^ percentile; quintile 3: 40^th^–<60^th^ percentile; quintile 4: 60^th^–<80^th^ percentile; quintile 5: ≥80^th^ percentile.

## Data Availability

The datasets used and analyzed during the current study are available from the corresponding author on request.
